# Toll interacting protein gene polymorphisms in patients with systemic sclerosis: association with interstitial lung disease, outcome, and survival

**DOI:** 10.3389/fmed.2025.1584014

**Published:** 2025-05-22

**Authors:** Niels Schröder, Jitka Andrä, Diana Knuth-Rehr, Silke Leja, Nicolas Hunzelmann, Pia Moinzadeh, Konrad Frank, Eda Börner, Francesco Bonella

**Affiliations:** ^1^Center for Interstitial and Rare Lung Diseases, Ruhrlandklinik, Pneumonology Department, University of Duisburg-Essen, Essen, Germany; ^2^Department of Dermatology and Venereology, University Hospital Cologne, Cologne, Germany; ^3^Department III of Internal Medicine, Section Pneumology, University Hospital Cologne, Cologne, Germany

**Keywords:** *TOLLIP*, systemic sclerosis, ILD, genetic variants, outcome

## Abstract

**Background:**

Pulmonary fibrosis is a leading cause of death in patients with Systemic sclerosis (SSc). Single nucleotide polymorphisms (SNPs) within Toll interacting protein (*TOLLIP*) coding gene have been associated with progression and prognosis of Idiopathic Pulmonary Fibrosis (IPF). Aim of the present study was to investigate the association of *TOLLIP* SNPs with the presence, severity and outcome of interstitial lung disease (ILD) in patients with SSc.

**Patients and methods:**

106 consecutive SSc patients (77 female) with (*N* = 53) and without ILD (*N* = 53) and 212 healthy controls (HC) (154 female) were genotyped for two SNPs within *TOLLIP* (rs3750920, rs5743890) by using TaqMan™ SNP Genotyping Assay (Thermo Fischer Scientific, USA). Disease progression was defined as ≥ 10% relative decline in FVC% pred. or ≥ 5 to < 10% decline in relative FVC% pred. and 15% relative decline in DLCO% pred. From baseline.

**Results:**

The *TOLLIP* rs5743890 minor Allele (C) was more frequent in HC than in SSc patients (41% vs. 16%, *p* = 0.021). The homozygote alleles of rs5743890 were significantly overrepresented in SSc patients compared to HC (84% vs. 71%, *p* = 0.008). Among SSc patients with ILD, those carrying the rs5743890 T/C genotype had a tendentially worse survival (158 vs. 213 months, *p* = 0.162) and a significantly higher rate of disease progression (66% vs. 22%, *p* = 0.003) compared to homozygotes. The rs5743890 minor allele C was an independent predictor of progression after adjustment for a number of covariates (HR 4.29, 95% CI 1.48–12.48, *p* = 0.008). Moreover, the TC haplotype appeared to be an even stronger predictor of progression than rs5743890 alone (HR 7.71, 95% CI 1.79–33.12, *p* = 0.006).

**Conclusion:**

*TOLLIP* SNP rs5743890 genotype distribution seems to differ in SSc patients compared to HC. The rs5743890 heterozygote genotype and the TC haplotype may be associated with an increased risk of progression in patients with SSc-ILD.

## 1 Introduction

Systemic sclerosis, also known as scleroderma, is a complex, immune-mediated rheumatic disease affecting multiple organs. It is characterized by fibrosis of the skin and internal organs but has a wide range of manifestations such as Raynaud’s phenomenon, vasculopathy and immune dysfunction (1). Amid all rheumatic diseases, Systemic sclerosis has the highest mortality associated with pulmonary involvement: interstitial lung disease (ILD), observed in around 80% of patients, and pulmonary hypertension (2).

Various risk factors are known to be associated with disease progression and mortality in patients with SSc-ILD like decline in forced vital capacity (FVC) and diffusing capacity of the lung for carbon monoxide (DLco) over 1 to 2 years. Older age, shorter disease duration, male sex, diffuse cutaneous systemic sclerosis (dcSSc) and positive anti-topoisomerase (anti-Scl-70) status are also risk indicating predictors of both progression and mortality (3–5). However, the challenge to predict progression and mortality in SSc still remains (6).

*TOLLIP* is a multifunctional intracellular protein that plays a significant role in innate immunity by preventing excessive pro-inflammatory responses (7). First described as an inhibitory adapter protein in the IL-1ß signaling pathway, research showed that *TOLLIP* mainly works as an inhibitor of the toll-like receptors (TLRs) 2 and 4 leading to a suppression of IL-6, IL-13, tumor necrosis factor-a (TNF-a) and transforming growth factor (TGF)-ß production (8, 9). Its inhibitory functions mostly rely on preventing IL-1 receptor-associated kinase-1 (IRAK-1) autophosphorylation and promoting receptor degradation which results in a down regulation of nuclear factor (NF)-κB activation (10).

Many genome-wide association studies (GWAS) linked *TOLLIP* SNPs to different diseases, especially IPF and rheumatoid arthritis (RA) associated interstitial lung disease (ILD) (11–14). These SNPs have been shown to affect the mRNA expression of *TOLLIP*, either increasing or lowering *TOLLIP* serum concentrations. The minor allele (C) of rs5743890 is associated with a 20% decrease of *TOLLIP* mRNA levels in lung tissue (11). Carriers of the rs3750920 minor allele also have a different response to treatment in IPF (15, 16).

The aim of this exploratory, single-center study was to investigate the association of *TOLLIP* SNPs with the presence, severity and outcome of ILD in patients with SSc.

## 2 Methods

### 2.1 Study subjects

We retrospectively studied 106 SSc patients (77 female) with (*N* = 53) and without (*N* = 53) interstitial lung disease. The patients were recruited at the University Hospital Cologne from 2004–2022. Diagnosis of Systemic Sclerosis was made according to the ACR-EULAR Criteria 2013 (1). Presence of ILD was diagnosed according to the most recent ATS/ERS guidelines (17).

The 212 healthy subjects (HC) (154 female) were recruited at the University Hospital Essen. These healthy controls between the ages of 30 and 70 years did not suffer from any lung disease and did not show any history of cancer, autoimmune diseases or immunodeficiency.

All included subjects provided written informed consent.

### 2.2 Clinical definitions

Disease progression of ILD was defined as either ≥ 10% relative decline in FVC% predicted or ≥ 5 to < 10% decline in relative FVC% predicted and 15% relative decline in DLCO% predicted (18). Otherwise, the patients were defined as stable. We did not consider death in the definition of progression since it was not possible to retrieve the cause of death (respiratory vs. nonrespiratory).

Time to progression was calculated from the first available lung function test to the timepoint when progression criteria were fulfilled. Patients without at least two follow-up lung function assessments were excluded from progression analysis. Survival was defined as the time from date of first hospital admission to the date of death or last available follow-up.

Disease duration was defined as timeframe between diagnosis of SSc and blood sampling.

The follow-up time was calculated from the first registered hospital admission (baseline).

### 2.3 DNA extraction and genotyping

Genomic DNA was extracted from peripheral whole blood samples by using a silica-membrane-based nucleic acid purification kit (QIAamp® Blood Mini Kit, Qiagen, USA), and stored at − 80°C before use. Two SNPs at the chr11p15.5 locus were chosen for this analysis: rs3750920 (position chr11:1309956) and rs5743890 (position chr11:1304599). Both SNPs are located in intronic regions.

Genotyping of the *TOLLIP* rs3750920 and rs5743890 SNP was performed by using TaqMan™ SNP Genotyping Assay (Thermo Fischer Scientific, Waltham, USA) and Applied Biosystems QuantStudio 3 RT-PCR System (Thermo Fischer Scientific, Waltham, USA) according to protocol.

### 2.4 Pulmonary function tests

Measurements included forced vital capacity (FVC), diffusion capacity of the lung for carbon monoxide (DLco) and the forced expiratory volume in one second (FEV1). Pulmonary function tests have been conducted at the Pulmonology Department of the University Hospital Cologne. Values were expressed as percentages of predicted normal values, based on the Global Lung Function Initiative (GLI) references (19). Lung function measurements shown in the manuscript are based on first available data.

### 2.5 Statistical analysis

Normal distribution for continuous variables was evaluated using the Kolmogorov–Smirnov test. Parametric data are presented as mean ± standard deviation (SD). Categorical variables are presented as either a percentage of the total or numerically. Continuous variables were analyzed with the unpaired t-test when comparing two groups. ANOVA was used to determine correlations when comparing more than two groups. To compare categorical variables, Chi-square test and Fisher’s exact test were used. Spearman’s or Pearson’s correlation coefficient was calculated to assess correlations. Allele, genotype and haplotype frequencies were calculated by using SNPStats (Catalan Institute of Oncology, Spain). Pair-wise linkage disequilibrium between both SNPs was also calculated by using SNPStats. Chi-square test and Fisher’s exact test were used to test for deviation from Hardy–Weinberg equilibrium (HWE).

Univariate and multivariable Cox proportional hazard regression were performed to examine the individual impact of age, gender, smoking history, medication, pulmonary hypertension, *TOLLIP* genotypes and pulmonary function tests on the disease course.

To determine a possible association between SNP genotypes and disease outcome, the Kaplan–Meier method with the log-rank test was used. *p* values < 0.05 were considered statistically significant. Survival was calculated from the time of first admission to the hospital.

All statistical analyses were performed using SPSS 29.0 (IBM, New York, USA) provided by the University of Duisburg-Essen.

## 3 Results

### 3.1 Characteristics of study subjects

Demographics and clinical characteristics of the studied subjects are shown in Table 1. Systemic sclerosis subtypes and specific SSc-related characteristics of patients are shown in Supplementary Tables S1, S2. At baseline, out of 106 SSc patients, 21 (20%) received antifibrotic treatment and 52 (50%) Immunosuppressive treatment, ten patients (9%) had no specific treatment for SSc (Supplementary Table S3). Further medications are also shown in Supplementary Table S3.

**Table 1:** Demographics and characteristics of the studied subjects at baseline.

Variable	All SSc (*N* = 106)	SSc-Non-ILD (*N* = 53)	SSc-ILD (*N* = 53)	HC (*N* = 212)
Age, years	60 ± 12.7^#^	60 ± 13.6	60 ± 11.9	56 ± 9.5
Gender, female	77 (73%)	43 (81%)	34 (64%)	154 (73%)
BMI, kg/m^2^*	24^#^ (21.6–26.4)	23.6 (21.5–25.7)	24.3 (22.1–27.5)	26 (23.8–29)
Smoking history, yes/no	40/66^#^	19/34	21/32	9/203
Pack years, *N*	27 ± 22	32 ± 26	20 ± 10	17 ± 6
FVC, % pred.	86 ± 22	100 ± 17 ^	73 ± 17	-
FEV1, % pred.	84 ± 20	95 ± 14 ^	72 ± 18	-
DLco, % pred.	65 ± 19	72 ± 17 ^	58 ± 19	-
Antifibrotic treatment, yes	21 (20%)	0 (0%) ^	21 (40%)	-
Immunosuppressive treatment, yes	52 (50%)	15 (28%) ^	36 (69%)	-
CRP, mg/dl*	0.24 (0.1–0.6)	0.13 (0.08–0.29)	0.38 (0.2–0.94)	-
Time to progression, months*	-	-	92 (53–148)	-
Time to death or loss to follow-up, months*	101 (53–157)	84 (26–149)	130 (71–176)	-

SSc, Systemic sclerosis; ILD, Interstitial lung disease; HC, Healthy controls; BMI, Body Mass Index; FVC, Forced vital capacity; FEV1, Forced expiratory volume in one second; DLco, Diffusion capacity of the lung for carbon monoxide; CRP, C-reactive protein. Otherwise indicated, values are expressed as mean ± standard deviation. ^*^Median, Interquartile range (IQR). ^#^*p* < 0.01 vs. HC. ^ *p* < 0.001 vs. SSc-ILD.

The median follow-up time of SSc patients was 101 (IQR: 53–157) months.

### 3.2 *TOLLIP* SNPs allele, haplotypes, and genotypes distribution

Allele distribution for both SNPs in SSc patients and HC is shown in Supplementary Table S4. *TOLLIP* rs3750920 and rs5743890 alleles were in Hardy–Weinberg equilibrium (HWE), except for the rs5743890 SSc-Non-ILD subgroup. The frequency of the rs5743890 minor allele C (0.16) in our healthy controls aligns with the distribution in a larger European cohort (11). The rs5743890 minor allele (C) was significantly overrepresented in HC than in SSc patients (*p* = 0.021), while no difference was observed in the frequency of the minor allele (T) of rs3750920 between SSc patients and HC (*p* = 0.242) (Supplementary Table S4). The best-fitting inheritance model for the association of rs5743890 with SSc was overdominant with overrepresentation of the rs5743890 homozygote genotypes in SSc patients compared to HC (Table 2). Three major haplotypes (frequency > 1%) were identified. The haplotype CT (rs3750920 C allele with rs5743890 T allele) occurred more frequently and TC (rs3750920 T allele with rs5743890 C allele) less frequently in SSc patients compared to HC (Supplementary Table S5).

**Table 2:** *TOLLIP* genotype distribution models in the studied subject.

*TOLLIP* rs3750920					
Model	Genotype	All SSc (*N* = 106)	HC (*N* = 212)	OR (95% CI)	*p* value
Codominant	C/C	39 (37%)	66 (31%)	1.00	0.43
	C/T	47 (44%)	94 (44%)	0.85 (0.50–1.44)	
	T/T	20 (18%)	52 (25%)	0.65 (0.34–1.25)	
Dominant	C/C	39 (37%)	66 (31%)	1.00	0.31
	C/T–T/T	67 (63%)	146 (69%)	0.78 (0.48–1.27)	
Recessive	C/C -C/T	86 (81%)	160 (76%)	1.00	0.25
	T/T	20 (19%)	52 (24%)	0.72 (0.40–1.28)	
Overdominant	C/C-T/T	59 (56%)	118 (56%)	1.00	1
	C/T	47 (44%)	94 (44%)	1.00 (0.63–1.60)	
Log-additive				0.81 (0.59–1.12)	0.2

*TOLLIP* rs5743890					
Model	Genotype	All SSc (*N* = 106)	HC (*N* = 212)	OR (95% CI)	*p* value
Codominant	T/T	87 (82%)	148 (70%)	1.00	0.027
	T/C	17 (16%)	62 (29%)	0.47 (0.26–0.85)	
	C/C	2 (2%)	2 (1%)	1.70 (0.24–12.29)	
Dominant	T/T	87 (82%)	148 (70%)	1.00	0.016
	T/C-C/C	19 (18%)	64 (30%)	0.51 (0.28–0.90)	
Recessive	T/T–T/C	104 (98%)	210 (99%)	1.00	0.49
	C/C	2 (2%)	2 (1%)	2.02 (0.28–14.54)	
Overdominant	T/T-C/C	89 (84%)	150 (71%)	1.00	0.008
	T/C	17 (16%)	62 (29%)	0.46 (0.25–0.84)	
Log-additive				0.58 (0.34–0.99)	0.04

SSc, Systemic sclerosis; HC, Healthy controls; OR, Odds ratio; CI, Confidence interval.

*TOLLIP* rs3750920 and rs5743890 were in high linkage disequilibrium (D′ 0.9992, r^2^ 0.1948, *p* < 0.0001).

### 3.3 *TOLLIP* SNPs and correlation with clinical characteristics

Table 3 shows the characteristics of SSc patients in relation to *TOLLIP* rs5743890 genotype. We did not observe any difference in age, gender, BMI and smoking history according to genotype and no association with the presence of ILD was seen (data not shown).

**Table 3:** Clinical characteristics of SSc patients at baseline stratified by rs5743890 genotype.

Variable	T/T (*N* = 87)	T/C (*N* = 17)	C/C (*N* = 2)	*p* value
Age, years	60 ± 13.0	61 ± 12.3	64 ± 10.0	0.802
Gender, female	67 (77%)	9 (53%)	1 (50%)	0.097°
BMI, kg/m^2^	24.2 ± 4.0	25.4 ± 4.2	25.2 ± 1.3	0.528
Smoking history, yes/no	29/58	10/7	1/1	0.131°
Pack years, (*N* = 21)	29 ± 21	13 ± 7	72 ± 0	0.027
FVC, % pred.	88 ± 21	76 ± 23	103 ± 13	0.081
FEV1, % pred.	84 ± 20	79 ± 18	95 ± 18	0.451
DLco, % pred.	66 ± 19	58 ± 19	84 ± 23	0.108
FVC decline, % pred./year	−1.8 ± 4.9	−1.4 ± 5.7	−1.3 ± 0.4	0.955
FEV1 decline, % pred./year	−1.2 ± 3.8	−2.8 ± 5.9	−0.3 ± 1	0.345
DLco decline, % pred./year	−1.3 ± 4.4	−2.3 ± 4.3	−3 ± 1.7	0.629
PAH, yes	23 (26%)	7 (41%)	0 (0%)	0.312°
Antifibrotic treatment, yes	19 (22%)	2 (12%)	0 (0%)	0.444°
Immunosuppressive treatment, yes	41 (48%)	10 (59%)	0 (0%)	0.268°
CVD, yes	10 (12%)	4 (24%)	0 (0%)	0.349°
ILD, yes	42 (48%)	11 (65%)	0 (0%)	0.167°

SSc, Systemic sclerosis; BMI, Body Mass Index; FVC, Forced vital capacity; FEV1, Forced expiratory volume in one second; DLco, Diffusion capacity of the lung for carbon monoxide; PAH, Pulmonary arterial hypertension; CVD, Cardiovascular diseases; ILD, Interstitial lung disease. Otherwise indicated, values are expressed as mean ± standard deviation. °Calculated by using Chi-Square test.

Patients with T/C genotype had a tendentially lower FVC % pred. and DLco % pred. at baseline than those carrying other genotypes but no differences related to the intake of immunosuppressive or antifibrotic drugs were seen.

### 3.4 Survival analysis

Median survival of all SSc patients was 101 (IQR: 53–157) months, 130 (IQR: 71–176) months in SSc-ILD patients and 84 (IQR: 26–149) months in those without ILD. Nine (9%) patients died until end of follow-up. No deaths occurred among SSc-Non-ILD patients and among those with ILD who remained stable.

Supplementary Figure S1 shows the survival curve of all SSc patients stratified for both rs3750920 and rs5743890 genotype. No significant differences were observed.

At KM analysis, a tendentially shorter survival was seen in SSc-ILD patients carrying rs5743890 T/C genotype compared to those with T/T genotype (158 vs. 213 months, *p* = 0.162) (Supplementary Figure S2). This tendency was confirmed when comparing the homozygous genotypes with the T/C genotype (Supplementary Figure S3).

### 3.5 ILD progression analysis

Disease progression occurred in 30 (57%) out of 53 patients with SSc-ILD at baseline. Median time to progression was 92 months (IQR: 53–148). There were no differences in terms of demographics and clinical characteristics between patients who had progression or those who remained stable at baseline (data not shown).

**Figure 1:**
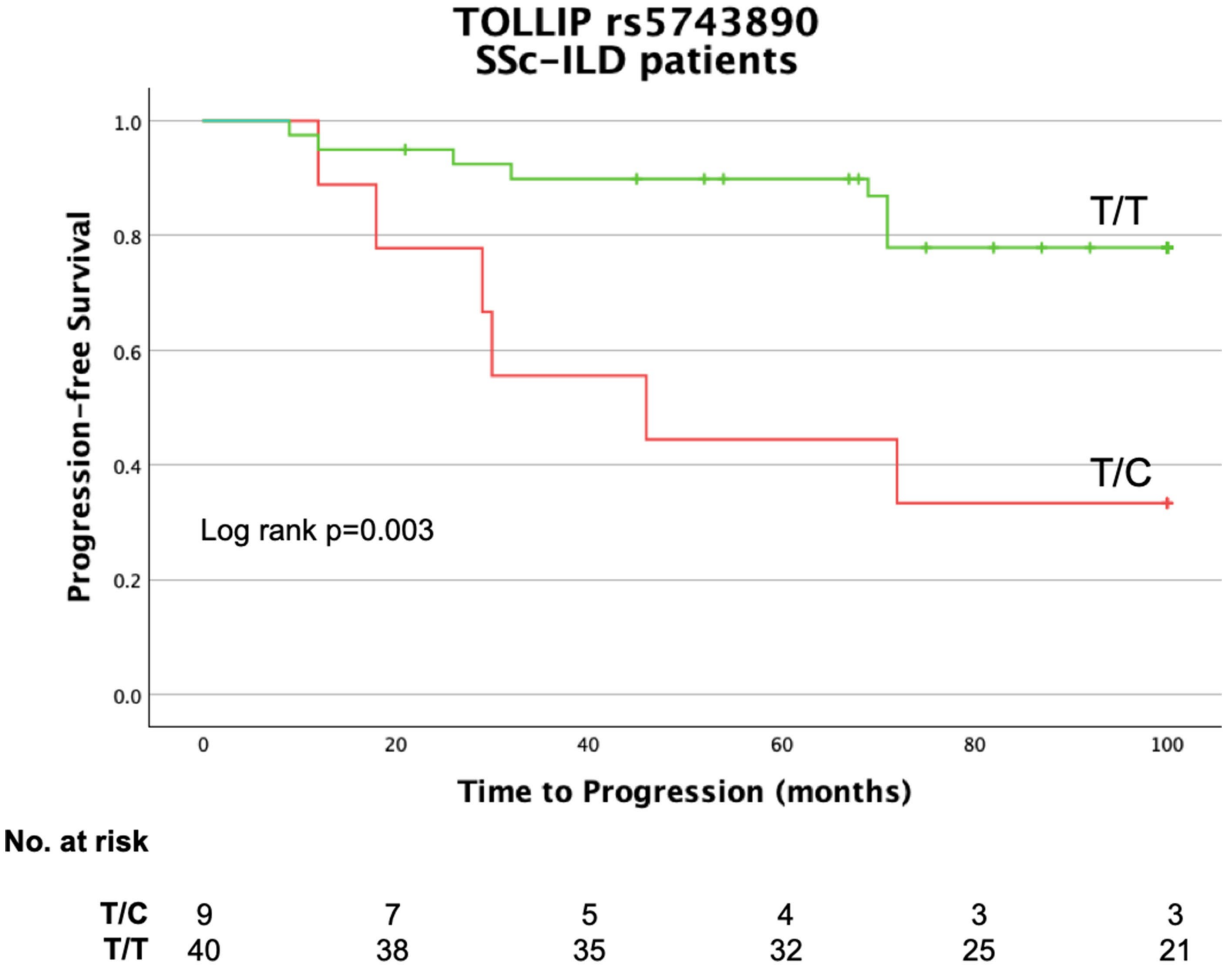
Kaplan–Meier curve for progression in SSc-ILD patients stratified by *TOLLIP* SNP rs5743890 genotype.

Patients carrying the *TOLLIP* rs5743890 minor allele C had a higher rate of disease progression than those who were homozygotes (66% vs. 22%, *p* = 0.003, respectively) (Figure 1). Disease progression rate did not vary according to *TOLLIP* rs3750920 genotype (Supplementary Figure S4).

In an additional exploratory analysis, we evaluated a combined endpoint of ILD progression and death. The presence of the *TOLLIP* rs5743890 minor allele C was associated with a significantly higher event rate compared to the T/T genotype (60% vs. 30%, *p* = 0.046, respectively) (Supplementary Figure S5).

### 3.6 Univariate and multivariable analysis for predictors of survival and progression

We conducted univariate and multivariable analysis using Cox regression to identify predictors of survival. We did not find any association between *TOLLIP* SNPs genotype and survival (data not shown). Additionally, we did not find any association of TC haplotype with survival (data not shown).

For progression, we performed univariate and multivariable Cox regression to assess predictors of ILD progression. The rs5743890 minor allele C was an independent predictor of progression after adjustment for a number of covariates (HR 4.29, 95% CI 1.48–12.48, *p* = 0.008) (Table 4).

**Table 4:** Univariate and multivariable Cox regression for predictors of progression in patients with SSc-ILD.

Variables	*β*	HR	95% CI	*p* value
Univariate analysis				
Age, years (continuous)	−0.019	0.98	0.90–1.07	0.660
Gender, (ref. male)	0.537	1.71	0.34–8.50	0.511
BMI, kg/m^2^ (continuous)	0.081	1.09	0.91–1.30	0.372
Smoking history, (ref. yes)	0.279	1.32	0.31–5.65	0.706
FVC, % pred. (continuous)	0.001	1.00	0.96–1.04	0.946
DLco, % pred. (continuous)	−0.033	0.97	0.93–1.01	0.103
*TOLLIP* rs5743890 (C allele), (ref. yes)	1.508	4.52	1.20–17.02	0.026
CVD, (ref. yes)	0.261	1.30	0.15–10.95	0.810
PAH, (ref. yes)	−0.567	0.57	0.12–2.71	0.477
Immunosuppressive treatment, (ref. yes)	−0.656	0.52	0.06–4.54	0.553
Steroids, (ref. yes)	0.435	1.55	0.38–6.26	0.543
Nintedanib, (ref. yes)	−0.065	0.94	0.28–3.19	0.917
Multivariable analysis*				
DLco, % pred. (continuous)	−0.027	0.97	0.94–1.00	0.084
*TOLLIP* rs5743890 (C allele), (ref. yes)	1.455	4.29	1.48–12.48	0.008

SSc-ILD, Systemic sclerosis-associated interstitial lung disease; HR, Hazard ratio; CI, Confidence interval; BMI, Body Mass Index; FVC, Forced vital capacity; FEV1, Forced expiratory volume in one second; DLco, Diffusion capacity of the lung for carbon monoxide; CVD, Cardiovascular diseases; PAH, Pulmonary arterial hypertension. ^*^Model obtained by using backward conditional stepwise regression (11 steps), including age, gender, BMI, Smoking history, FVC (% pred.), CVD, PAH, Immunosuppressive treatment, Steroids and Nintedanib as covariates.

For the sake of completeness, we performed a Cox regression analysis stratified by antifibrotic treatment, but we did not identify significant predictors, due to the low number of cases and events (data not shown).

Since 39 (74%) patients from the initial SSc-Non-ILD group (at baseline) developed ILD over time, we performed a KM analysis and Cox regression for progression in the entire SSc-ILD cohort.

At KM analysis, patients carrying the *TOLLIP* rs5743890 minor allele C had a significantly higher rate of disease progression than those with the T/T genotype (63% vs. 29%, *p* = 0.019) (Supplementary Figure S6). This finding was confirmed at multivariable Cox regression, where the rs5743890 minor allele C, was identified as a predictor of progression after adjustment for a number of covariates (HR 2.47, 95% CI 1.12–5.46, *p* = 0.025) (Supplementary Table S6).

In a further exploratory analysis, we examined the impact of the TC (rs3750920 T allele with rs5743890 C allele) haplotype on ILD progression. Kaplan–Meier analysis revealed a significantly higher rate of disease progression in carriers of the TC haplotype (60% vs. 25%, *p* = 0.039) (Supplementary Figure S7). Carriage of the TC haplotype was independently associated with a significantly increased risk of disease progression after adjustment for a number of covariates (HR 7.71, 95% CI 1.79–33.12, *p* = 0.006) (Supplementary Table S7).

## 4 Discussion

To the best of our knowledge, this is the first study evaluating a possible association of *TOLLIP* polymorphisms with SSc, the presence of ILD and outcome. The distribution of *TOLLIP* rs5743890 genotype was significantly different between SSc patients and HC, but not between patients with and without ILD. In addition, we found that the rs5743890 minor allele C was associated with ILD progression in patients with SSc-ILD.

*TOLLIP* is predominantly expressed in immune cells and alveolar type 1 epithelial cells (20), with reduced overall expression in the lung tissue of IPF patients (11). However, atypical epithelial cells in IPF lungs show an enhanced expression of *TOLLIP*, the significance of which is uncertain (14). Given the increasing evidence that a tight homeostatic regulation of *TOLLIP* may impact the development of pulmonary fibrosis, we selected two SNPs within the *TOLLIP* coding gene based on their previously reported association with IPF and their potential relevance in the pathogenesis of autoimmune diseases like RA (19).

In our analysis, we observed a high D′ value (0.9992) but a relatively low r^2^ value (0.1948) between the two SNPs. This discrepancy can be explained by the allele frequencies in the overall cohort, as shown in Supplementary Table S4. Although the D′ value suggests high linkage disequilibrium, the low r^2^ indicates that the SNPs are not perfectly correlated and may independently contribute to the observed phenotypes. This highlights the potential importance of considering both SNPs, as each may provide unique information.

We observed that the homozygote genotypes of *TOLLIP* rs5743890 were significantly overrepresented in SSc patients compared to HC, suggesting the T/C genotype to have a protective effect against SSc. Additionally, we identified a haplotype (TC) which was significantly less frequent in patients with SSc than HC (OR 0.57, *p* = 0.043).

However, we found that the dominant model was also significant (OR 0.51, *p* = 0.016). All in all, these findings should be interpreted with caution, given the limited sample size (Table 2).

Beside the possible association with SSc, we did not find any significant differences in the allele, haplotype and genotype distribution according to the presence or absence of ILD. This is in line with what has been already observed for MUC5B rs35705950 in SSc-ILD (21, 22). *TOLLIP* and MUC5B variants are both located on chromosome 11 and in very close proximity to each other, providing a possible explanation to the lack of association between both SNPs and ILD in SSc. Interestingly, while the minor allele of MUC5B rs35705950 is known to be the dominant risk factor for developing IPF (12, 23, 24), the minor allele of *TOLLIP* rs5743890 seems to have an opposing effect in our findings, acting protectively against SSc.

With regard to ILD progression, we found that the rs5743890 minor allele C was an independent predictor at multivariable analysis after adjustment for several covariates, including treatment. ILD patients carrying the minor allele had a significantly shorter time to disease progression and a tendentially worse survival than the homozygotes for the major allele. This finding aligns with the results of another study on *TOLLIP* in IPF, where the T/C genotype was associated to a significantly shorter time to disease progression compared to the T/T genotype (43.5 vs. 63 months) (12). On the other hand, we did not see any differences in the annual decline in FVC % pred. or DLco % pred. According to genotype (Table 3). We cannot exclude that the observed association of the genotype with ILD progression might be driven by differences in the distribution of HRCT patterns (UIP vs. NSIP, fibrotic vs. inflammatory) according to genotype, but we were not able to retrieve the CT scans of all SSc-ILD patients.

Whether *TOLLIP* genotype might be a reliable biomarker to stratify SSc patients for the risk of ILD progression needs to be verified in further studies. To support this finding, we also conducted an exploratory Kaplan–Meier analysis using a composite endpoint that included ILD progression and death. While this approach strengthened the observed significant association with the rs5743890 minor allele C (Supplementary Figure S5), we acknowledge that the exact cause of death could not be determined. As such, this combined outcome carries the risk of introducing classification bias and should be interpreted as exploratory.

Although the minor allele C in *TOLLIP* rs3750920 has been found to be associated with a poor response or even a trend toward harm for N-Acetylcysteine treatment in patients with IPF (15, 16), we did not observe any association with treatment in our cohort. A clinical trial using *TOLLIP* genotyping for N-Acetylcysteine selection in IPF is ongoing and will hopefully provide a definitive answer (Clinical trial number: not applicable).

Beyond single SNP effects, we also investigated the influence of haplotypic combinations of rs3750920 and rs5743890. Carriers of the TC haplotype exhibited a significantly higher risk of ILD progression (HR 7.71, 95% CI 1.79–33.12, *p* = 0.006). This finding supports the idea that also the combined occurrence of genetic variants in specific haplotypes may contribute to the course of ILD in SSc patients. In other fibrosing lung diseases, *TOLLIP* haplotypes have also been linked to disease susceptibility and survival. A *MUC5B–TOLLIP* haplotype was associated with reduced survival in fibrotic hypersensitivity pneumonitis (HR 6.92, *p* = 0.006), while in IPF, another *MUC5B–TOLLIP* haplotype appeared to be protective (HR 0.37, *p* = 0.009) (25, 26). These observations further highlight the potential relevance of *TOLLIP* haplotypes in fibrosing ILDs.

Notably, SSc-ILD patients showed a longer median survival compared to those without ILD. This paradoxical finding may be explained by earlier hospital contact and longer follow-up time in ILD patients, as well as by our endpoint definition, which combined death and loss to follow-up.

Although this study presents novel findings, it has several limitations. First, the monocentric nature of the study. Second, the sample size of SSc patients was too small to perform subgroup-analyses. Third, the absence of a replication cohort limits the generalizability of our results. Fourth, the lack of detailed information on the ethnic background of the studied subjects. While it is reasonable to assume that the majority of individuals in our cohort are of Caucasian ancestry, based on the recruitment setting, we cannot confirm this with certainty. Fifth, HRCT scans were not systematically collected so that we could not verify an association between HRCT pattern and SNPs. Sixth, given the retrospective design, lung function tests were not collected at the same time intervals for all patients, so that we had to normalize the results accordingly.

Seventh, the antifibrotic and immunosuppressive therapies varied considerably among patients with SSc-ILD, indicating a potential underuse of disease-modifying therapies. However, due to the retrospective design of the study, detailed data on treatment regimens, combinations or duration was unavailable. This may have confounded the observed associations and should be addressed in prospective studies.

Furthermore, in the present study we did not investigate the impact of *TOLLIP* genotypes on serum protein concentrations. These data may help to clarify functional implications and strengthen the biological relevance of the genetic findings presented here, and will be the object of a further study.

In conclusion, this study provided a new insight into the role of *TOLLIP* gene polymorphisms in SSc, with and without ILD. The minor allele C of *TOLLIP* rs5743890 and the TC haplotype are associated with reduced disease susceptibility, but seem to be independent risk factors for ILD progression in SSc. Additionally, the interaction between the two *TOLLIP* SNPs appears to be a stronger predictor of ILD progression than the rs5743890 minor allele C alone. Further validation with larger cohorts in a multi-center fashion is mandatory.

## Data Availability

The raw data supporting the conclusions of this article will be made available by the authors, without undue reservation.

## Glossary

Anti-Scl-70: Anti-topoisomerase-I antibodies
BMI: Body mass index
CI: Confidence interval
COPD: Chronic obstructive pulmonary disease
CRP: C-reactive protein
CVD: Cardiovascular diseases
dcSSc: Diffuse Cutaneous Systemic Sclerosis
DLco: Diffusion capacity of the lung for carbon monoxide
FEV1: Forced expiratory volume in one second
FVC: Forced vital capacity
GWAS: Genome-wide association studies
HC: Healthy controls
HWE: Hardy–Weinberg equilibrium
IL-x: Interleukin
ILD: Interstitial lung disease
IPF: Idiopathic pulmonary fibrosis
IQR: Interquartile range
IRAK-1: IL-1 receptor-associated kinase-1
lcSSc: Limited Cutaneous Systemic Sclerosis
NF-κB: Nuclear factor κB
PAH: Pulmonary arterial hypertension
RA: Rheumatoid arthritis
SNP: Single nucleotide polymorphisms
SSc: Systemic sclerosis
TGF- ß: Transcription growth factor ß
TLR: Toll-like receptors
TNF-a: Tumor necrosis factor-a
*TOLLIP*: Toll interacting protein
