# Supplementary material for: Toll interacting protein gene polymorphisms in patients with systemic sclerosis: association with interstitial lung disease, outcome, and survival
Source: Front Med (Lausanne). 2025 May 22;12:1584014. doi: 10.3389/fmed.2025.1584014 (PMC12137070; doi:10.3389/fmed.2025.1584014)
Supplement: Supplementary file 1 [file Data_Sheet_1.docx]

**SUPPLEMENTAL MATERIAL**

**TABLES**

**Table S1:** Systemic Sclerosis subtype distribution according to presence or absence of ILD at baseline.

|  | All SSc  (N=106) | SSc-Non-ILD (N=53) | SSc-ILD (N=53) | p value |
| --- | --- | --- | --- | --- |
| LcSSc | 49 (46) | 33 (62) | 16 (30) | < 0.001 |
| DcSSc | 34 (32) | 5 (9) | 29 (55) | < 0.001 |
| SSc sine scleroderma | 3 (3) | 0 (0) | 3 (6) | 0.079 |
| Other (not further classified) | 20 (19) | 15 (28) | 5 (9) | 0.013 |

Abbreviations: SSc: Systemic sclerosis; SSc-ILD: Systemic sclerosis-associated interstitial lung disease; LcSSc: Limited Cutaneous Systemic Sclerosis; DcSSc: Diffuse Cutaneous Systemic Sclerosis.

Otherwise indicated, values are expressed as N (%).

p value calculated by using Chi-Square test

**Table S2:** Distribution of SSc clinical characteristics according to presence or absence of ILD at baseline.

|  | All SSc (N=106) | SSc-Non-ILD (N=53) | SSc-ILD (N=53) | p value |
| --- | --- | --- | --- | --- |
| Organ Involvement  None  Esophagus  Stomach  Heart  Kidneys  Musculoskeletal System  Skin | 53 (51) 77 (73) 32 (30) 17 (16) 10 (9) 36 (34)  79 (75) | 38 (72) 34 (64) 10 (19) 2 (4) 6 (11) 11 (21)  33 (62) | 16 (30) 43 (81) 22 (42) 15 (28) 4 (8) 25 (47)  46 (87) | < 0.001  0.05  0.011  < 0.001  0.506  < 0.01  < 0.01 |
| Sicca Syndrome | 34 (32) | 5 (9) | 29 (55) | < 0.01 |
| Pulmonary Hypertension | 30 (28) | 8 (15) | 22 (42) | < 0.01 |
| History of Cancer | 13 (12) | 8 (15) | 5 (9) | 0.374 |

Abbreviations: SSc: Systemic sclerosis; SSc-ILD: Systemic sclerosis-associated interstitial lung disease.

Otherwise indicated, values are expressed as N (%).

p value calculated by using Chi-Square test

**Table S3:** Distribution of medication according to presence or absence of ILD at baseline.

|  | All SSc (N=106) | SSc-Non-ILD (N=53) | SSc-ILD (N=53) | p value |
| --- | --- | --- | --- | --- |
| Analgetics | 47 (44) | 22 (42) | 25 (47) | 0.558 |
| Antihypertensive Medication  ACE-Inhibitors  Angiotensin Receptor Blockers  Beta-Blockers  Calcium Channel Blockers | 42 (40) 35 (33) 30 (28) 58 (55) | 17 (32) 14 (26) 12 (23) 29 (55) | 25 (47) 21 (40) 18 (34) 29 (55) | 0.112  0.148  0.196  1 |
| ASS | 31 (29) | 11 (21) | 20 (40) | 0.055 |
| Immunosuppressive Therapy  Cyclophosphamid  Methotrexat  Azathioprin  Mycophenolat-Mofetil  Chloroquin  Other | 8 (8) 22 (21) 4 (4) 11 (10) 6 (6) 12 (11) | 1 (2) 9 (17) 2 (4) 2 (4) 2 (4) 4 (8) | 7 (13) 13 (25) 2 (4) 9 (17) 4 (8) 8 (15) | 0.027  0.338  1  0.026  0.401  0.220 |
| Nintedanib | 21 (20) | 0 (0) | 21 (40) | < 0.001 |
| PDE5-Inhibitors  Sildenafil  Tadalafil  Unknown | 8 (8) 9 (9) 12 (11) | 3 (6) 5 (9) 0 (0) | 5 (9) 4 (8) 12 (22) | 0.462  0.727  < 0.001 |
| Prostanoids  Iloprost  Prostavasin | 69 (65) 1 (1) | 28 (53) 1 (2) | 41 (77) 0 (0) | < 0.01  0.315 |
| Proton Pump Inhibitors | 92 (87) | 42 (79) | 50 (94) | 0.022 |
| sGC-Stimulators | 13 (12) | 2 (4) | 11 (21) | < 0.01 |
| Steroids | 43 (41) | 11 (21) | 32 (62) | < 0.001 |

Abbreviations: SSc: Systemic sclerosis; SSc-ILD: Systemic sclerosis-associated interstitial lung disease; ACE: Angiotensin-converting enzyme; PDE5: Phosphodiesterase-5; sGC: Soluble guanylyl cyclase; ASS: acetylsalicylic acid.

Values are expressed as N (%).

p value calculated by using Chi-Square test.

**Table S4**: Allele frequency for each SNP in the study cohort.

| rs3750920 (allele) |  |  |  |  |  |
| --- | --- | --- | --- | --- | --- |
|  | **All subjects (N=318)** | **All SSc (N=106)** | **SSc-Non-ILD (N=53)** | **SSc-ILD  (N=53)** | **HC  (N=212)** |
| **C** (%) | 351 (55) | 125 (59) | 68 (64) | 57 (54) | 226 (53) |
| **T** (%) | 285 (45) | 87 (41) | 38 (36) | 49 (46) | 198 (47) |
| **HWE (p value)** | 0.07 | 0.42 | 0.91 | 0.36 | 0.13 |

| rs5743890 (allele) |  |  |  |  |  |
| --- | --- | --- | --- | --- | --- |
|  | **All subjects (N=318)** | **All SSc (N=106)** | **SSc-Non-ILD (N=53)** | **SSc-ILD  (N=53)** | **HC  (N=212)** |
| **T** (%) | 549 (86) | 191 (90) | 96 (91) | 95 (90) | 358 (84) |
| **C** (%) | 87 (14) | 21 (10) | 10 (9) | 11 (10) | 66 (16) ^#^ |
| **HWE (p value)** | 0.48 | 0.26 | 0.01 | 0.4 | 0.12 |

Abbreviations: SSc: Systemic sclerosis; SSc-ILD: Systemic sclerosis-associated interstitial lung disease; HC: Healthy controls; HWE: Hardy-Weinberg equilibrium.

^#^ p=0.021 vs. SSc

**Table S5:** *TOLLIP* haplotype frequencies and association with SSc in the studied subjects (frequency > 1 %).

| Common haplotypes | SSc patients (freq.) | HC (freq.) | OR | 95% CI | p value |
| --- | --- | --- | --- | --- | --- |
| **CT** | 0.5896 | 0.533 | 1.00 | - | - |
| **TT** | 0.3113 | 0.3113 | 0.93 | 0.65 – 1.33 | 0.7 |
| **TC** | 0.0991 | 0.1557 | 0.57 | 0.33 – 0.98 | 0.043 |

Global haplotype association p value: 0.11

Abbreviations: SSc: Systemic sclerosis; HC: Healthy controls; OR: Odds ratio; CI: Confidence interval.

**Table S6**: Univariate and multivariable Cox regression of ILD progression in patients who developed ILD over time.

| **Variables** | **β** | **HR** | **95 % CI** | **p value** |
| --- | --- | --- | --- | --- |
| ***Univariate analysis*** |  |  |  |  |
| **Age,** years (continuous) | 0.025 | 1.03 | 0.98 – 1.08 | 0.331 |
| **Gender,** (ref. male) | -0.358 | 0.70 | 0.28 – 1.74 | 0.441 |
| **BMI,** kg/m^2^ (continuous) | 0.059 | 1.06 | 0.96 – 1.17 | 0.242 |
| **Smoking history,** (ref. yes) | 0.118 | 1.13 | 0.48 – 2.64 | 0.786 |
| **FVC,** % pred. (continuous) | 0.021 | 1.02 | 1.00 – 1.05 | 0.086 |
| **DLco,** % pred. (continuous) | -0.018 | 0.98 | 0.96 – 1.01 | 0.164 |
| **TOLLIP rs5743890 (C allele),** (ref. yes) | 0.831 | 2.30 | 1.00 – 5.25 | 0.049 |
| **CVD,** (ref. yes) | -0.075 | 0.93 | 0.29 – 2.93 | 0.898 |
| **PAH,** (ref. yes) | -1.206 | 0.30 | 0.09 – 0.97 | 0.044 |
| **Immunosuppressive treatment,** (ref. yes) | 0.332 | 1.39 | 0.55 – 3.53 | 0.485 |
| **Steroids,** (ref. yes) | 0.350 | 1.42 | 0.61 – 3.30 | 0.416 |
| **Nintedanib,** (ref. yes) | 0.120 | 1.13 | 0.45 – 2.82 | 0.798 |
| ***Multivariable analysis**** |  |  |  |  |
| **TOLLIP rs5743890 (C allele),** (ref. yes) | 0.905 | 2.47 | 1.12 – 5.46 | 0.025 |

Abbreviations: SSc-ILD: Systemic sclerosis-associated interstitial lung disease; HR: Hazard ratio; CI: Confidence interval; BMI: Body Mass Index; FVC: Forced vital capacity; FEV1: Forced expiratory volume in one second; DLco: Diffusion capacity of the lung for carbon monoxide; CVD: Cardiovascular diseases; PAH: Pulmonary arterial hypertension.

* Model obtained by using backward conditional stepwise regression (11 steps), including age, gender, BMI, Smoking history, FVC (% pred.), DLco (% pred.), CVD, PAH, Immunosuppressive treatment, Steroids and Nintedanib as covariates.

**Table S7**: Univariate and multivariable Cox regression for predictors of progression in patients with SSc-ILD, including the TC haplotype as a covariate.

| **Variables** | **β** | **HR** | **95 % CI** | **p value** |
| --- | --- | --- | --- | --- |
| ***Univariate analysis*** |  |  |  |  |
| **Age,** years (continuous) | -0.029 | 0.97 | 0.88 – 1.07 | 0.550 |
| **Gender,** (ref. male) | 0.511 | 1.67 | 0.34 – 8.23 | 0.530 |
| **BMI,** kg/m^2^ (continuous) | 0.021 | 1.02 | 0.86 – 1.21 | 0.814 |
| **Smoking history,** (ref. yes) | 0.271 | 1.31 | 0.32 – 5.41 | 0.708 |
| **FVC,** % pred. (continuous) | -0.004 | 1.00 | 0.96 – 1.04 | 0.855 |
| **DLco,** % pred. (continuous) | -0.041 | 0.96 | 0.92 – 1.00 | 0.043 |
| **TOLLIP TC haplotype,** (ref. yes) | 2.403 | 11.06 | 1.75 – 69.82 | 0.011 |
| **CVD,** (ref. yes) | 0.074 | 1.08 | 0.11 – 10.84 | 0.950 |
| **PAH,** (ref. yes) | -1.122 | 0.33 | 0.06 – 1.78 | 0.196 |
| **Immunosuppressive treatment,** (ref. yes) | -0.345 | 0.71 | 0.09 – 5.50 | 0.742 |
| **Steroids,** (ref. yes) | 0.720 | 2.05 | 0.49 – 8.56 | 0.323 |
| **Nintedanib,** (ref. yes) | -0.378 | 0.69 | 0.21 – 2.29 | 0.539 |
| ***Multivariable analysis**** |  |  |  |  |
| **DLco,** % pred. (continuous) | -0.033 | 0.97 | 0.94 – 1.00 | 0.050 |
| **PAH,** (ref. yes) | -1.073 | 0.34 | 0.10 – 1.19 | 0.092 |
| **TOLLIP TC haplotype,** (ref. yes) | 2.042 | 7.71 | 1.79 – 33.12 | 0.006 |

Abbreviations: SSc-ILD: Systemic sclerosis-associated interstitial lung disease; HR: Hazard ratio; CI: Confidence interval; BMI: Body Mass Index; FVC: Forced vital capacity; FEV1: Forced expiratory volume in one second; DLco: Diffusion capacity of the lung for carbon monoxide; CVD: Cardiovascular diseases; PAH: Pulmonary arterial hypertension.

* Model obtained by using backward conditional stepwise regression (10 steps), including age, gender, BMI, Smoking history, FVC (% pred.), DLco (% pred.), CVD, PAH, Immunosuppressive treatment, Steroids and Nintedanib as covariates.

**FIGURES**


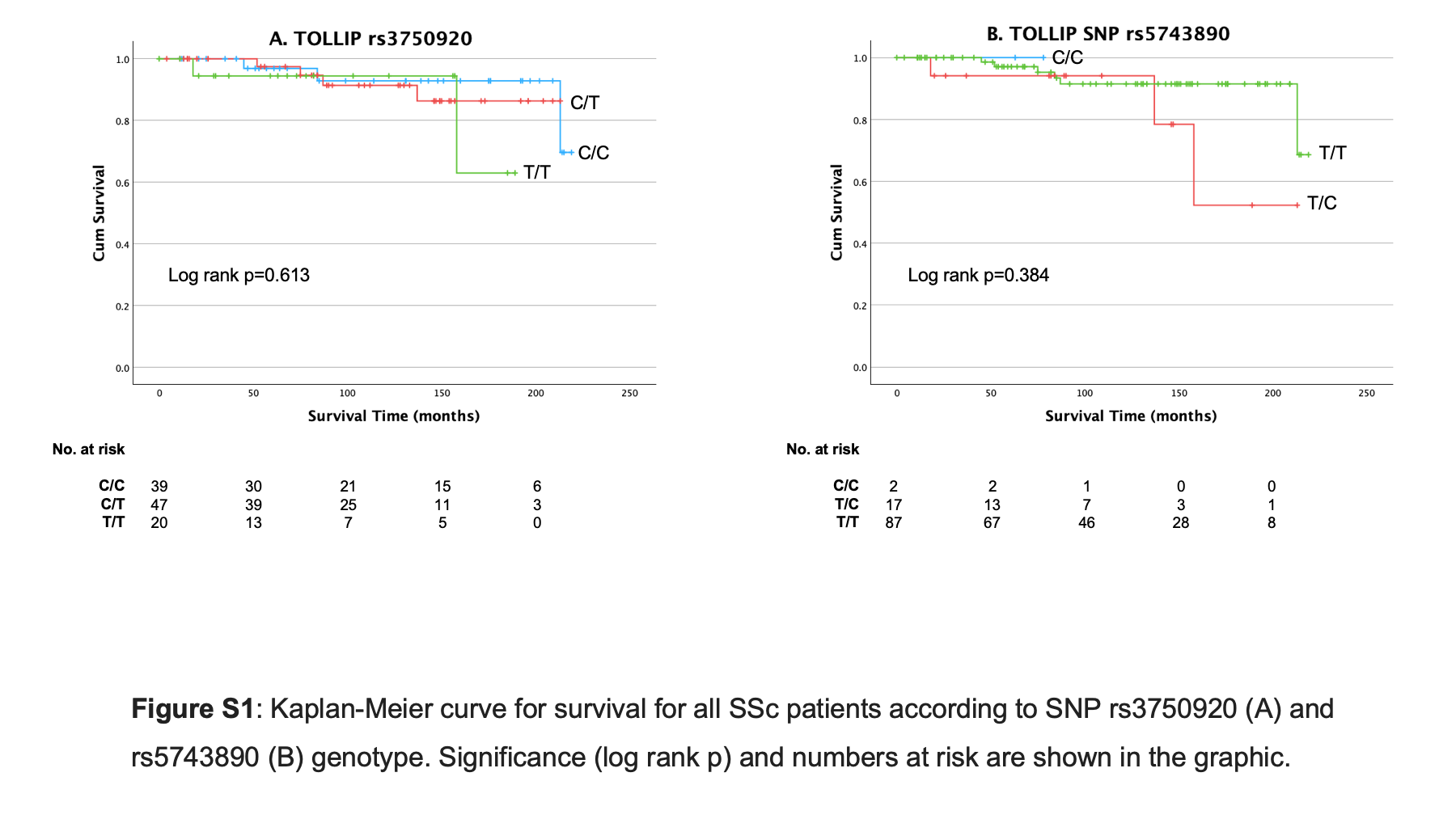


**Figure S1**: Kaplan-Meier survival curves for SSc patients according to SNP rs3750920 (A) and rs5743890 (B) genotype.


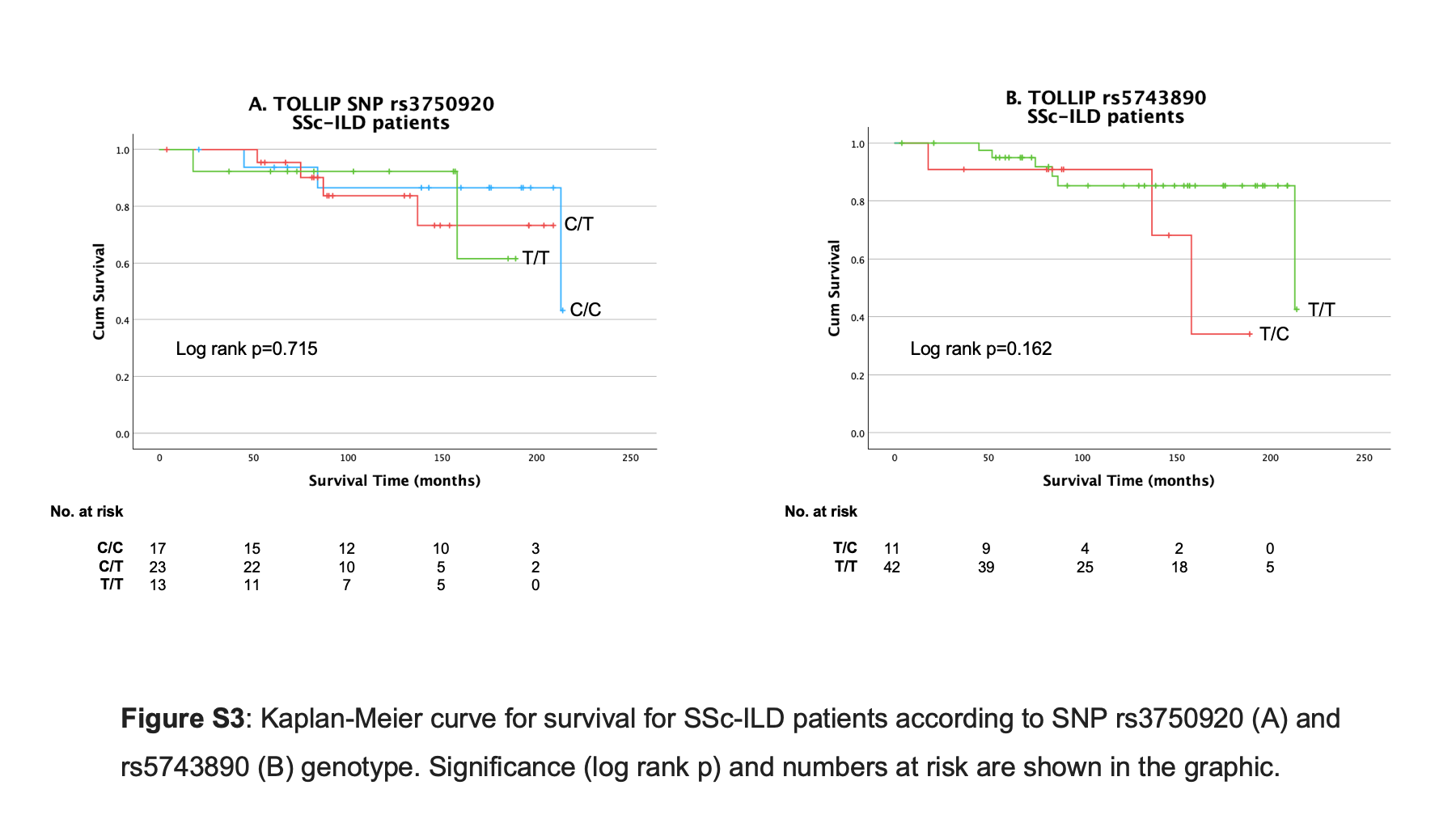

**Figure S2**: Kaplan-Meier survival curves for SSc-ILD patients according to SNP rs3750920 (A) and rs5743890 (B) genotype.


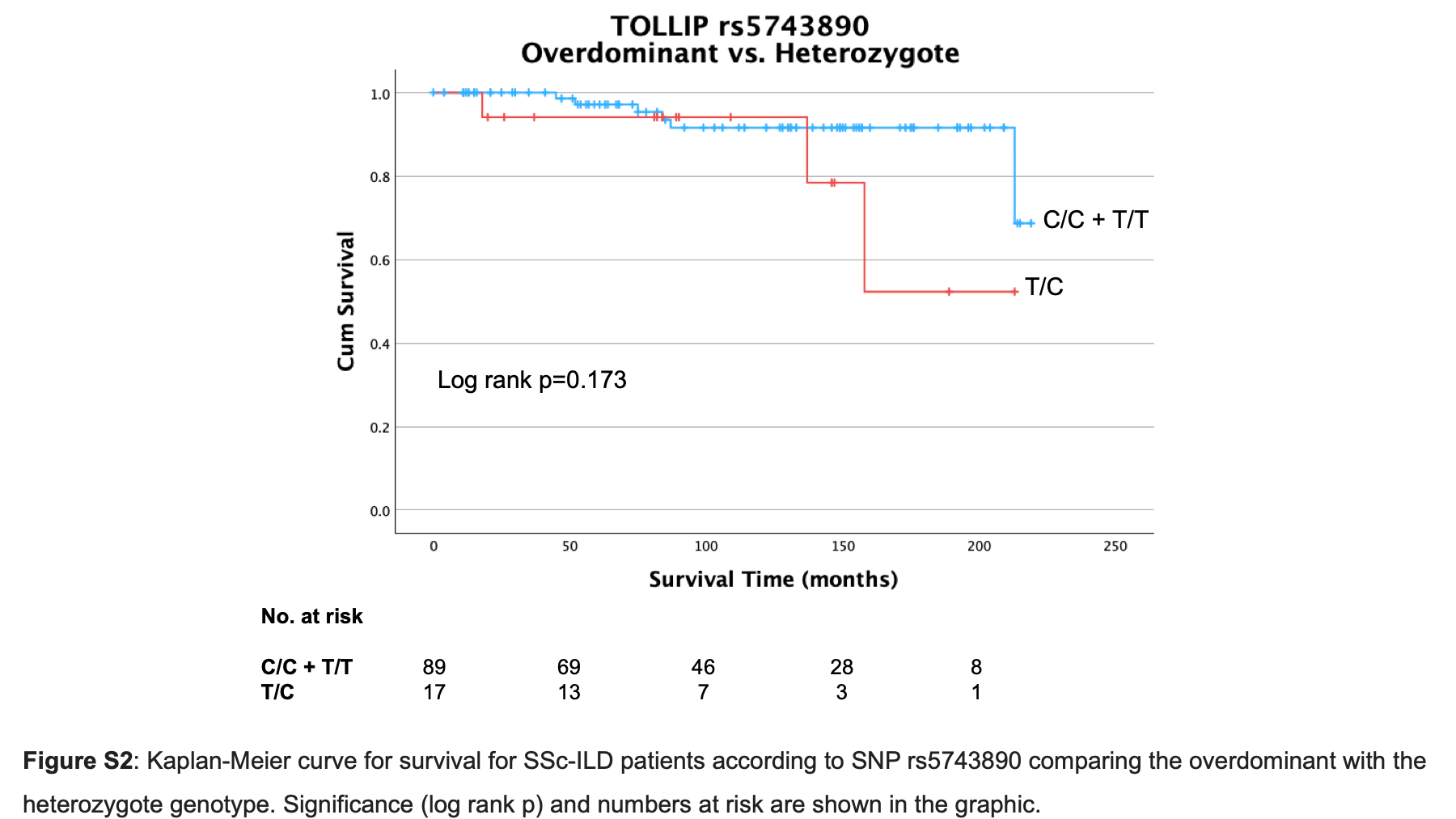


**Figure S3**: Kaplan-Meier survival curve for SSc patients comparing overdominant and heterozygote genotypes according to SNP rs5743890.


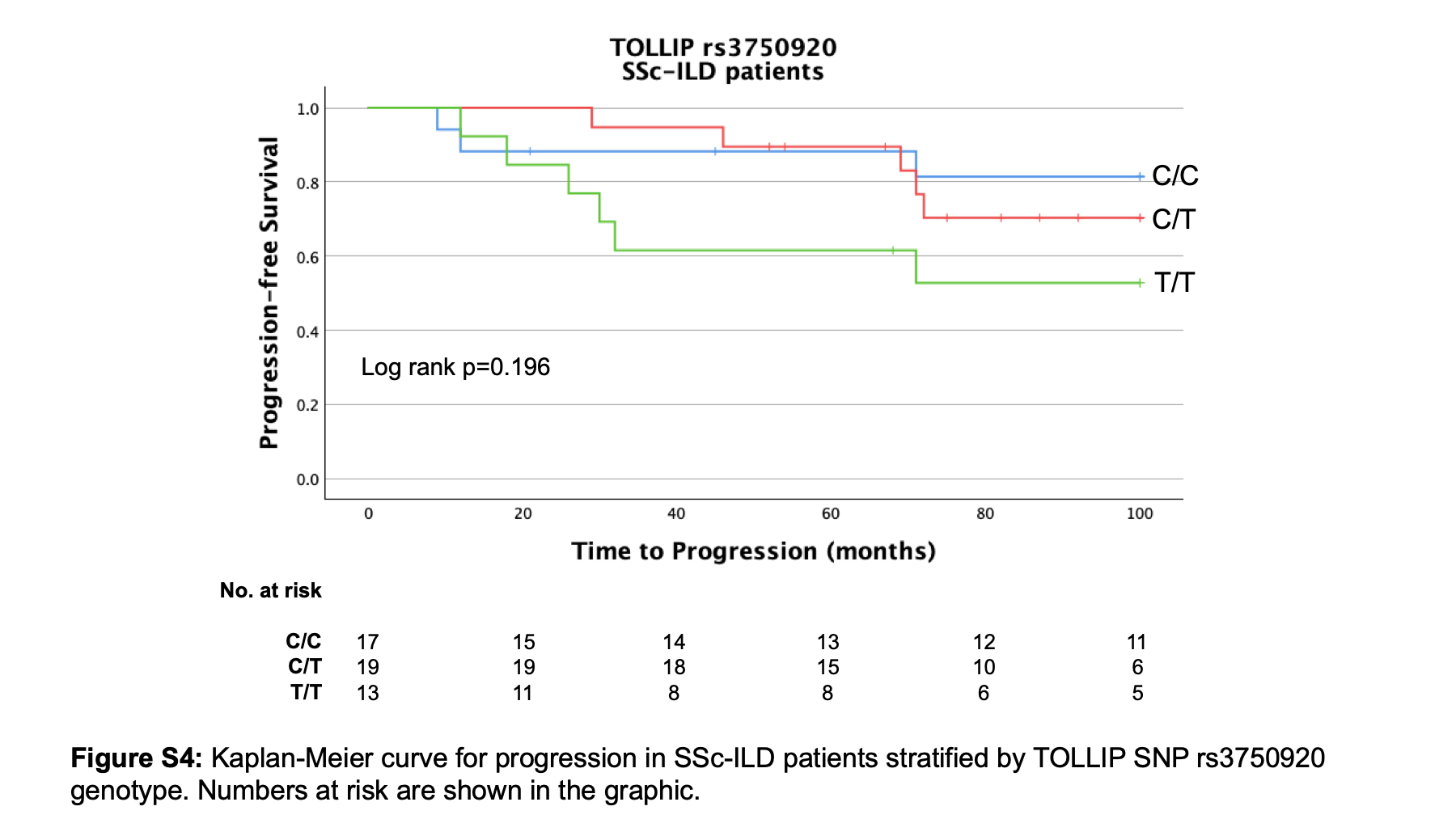


**Figure S4**: Kaplan-Meier curve for progression in SSc-ILD patients stratified by TOLLIP SNP rs3750920 genotype.


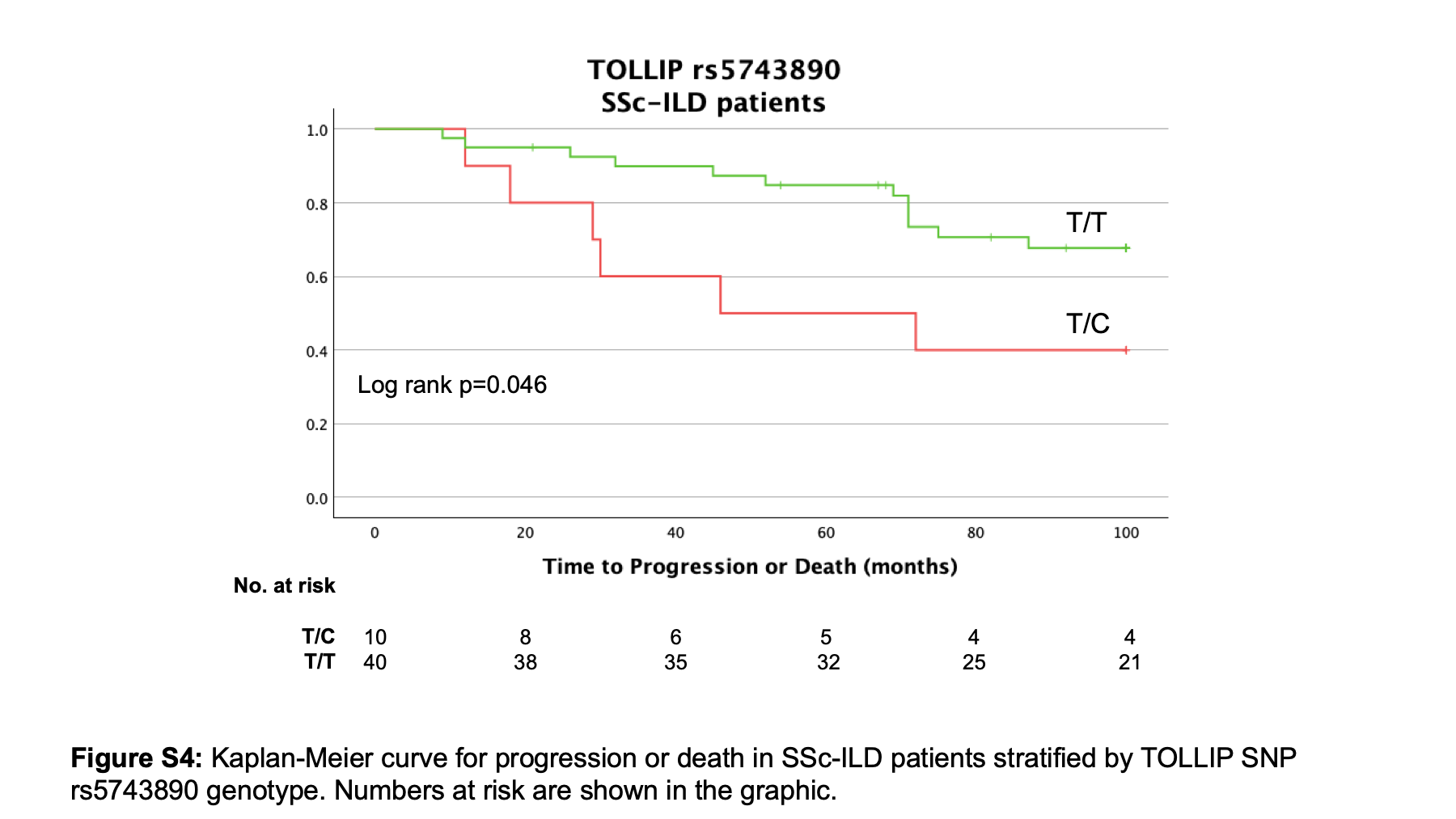


**Figure S5:** Kaplan-Meier curve for progression or death in SSc-ILD patients stratified by TOLLIP SNP rs5743890 genotype.


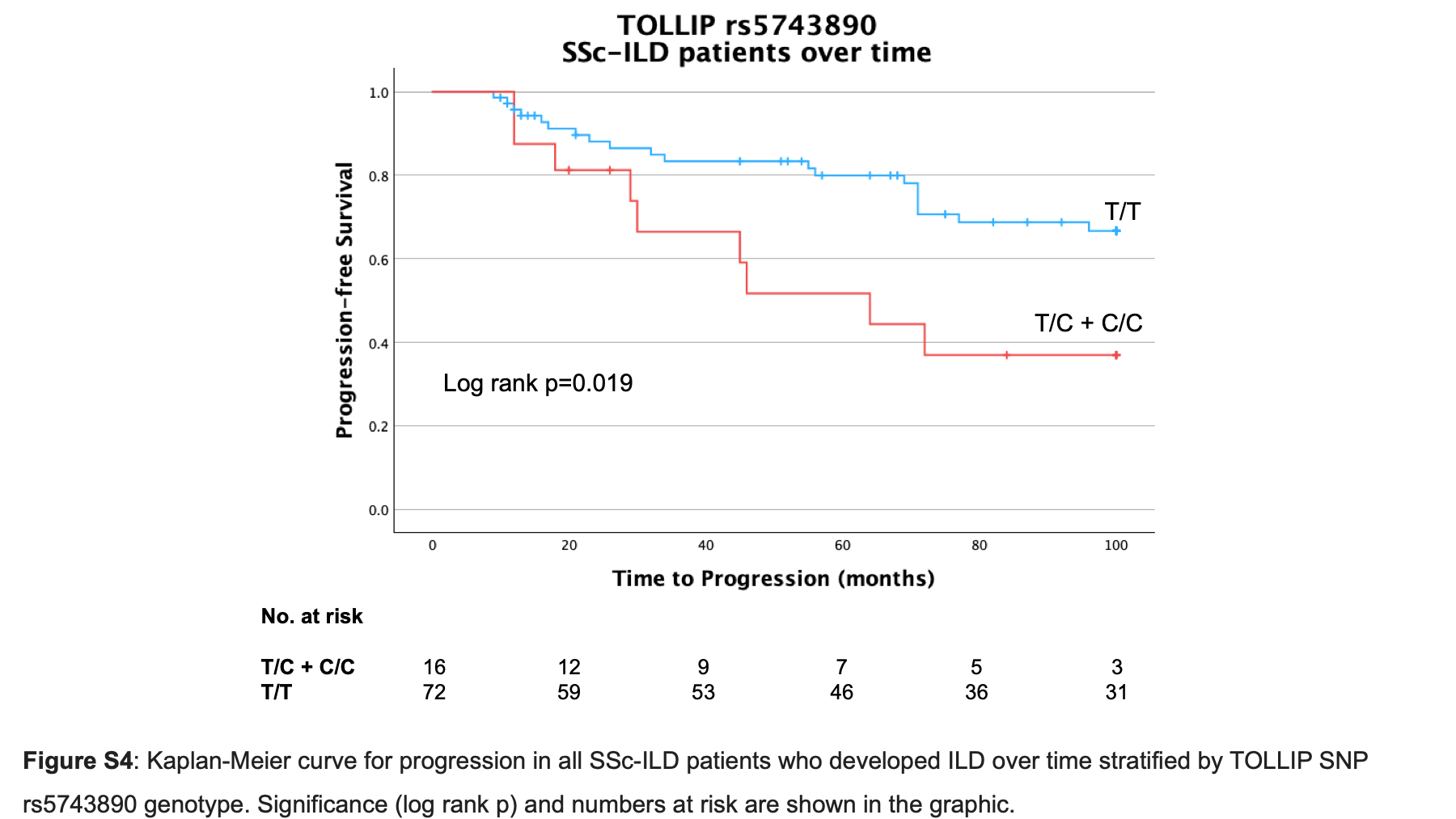


**Figure S6**: Kaplan-Meier curve for ILD progression who developed ILD over time stratified by the presence of TOLLIP SNP rs5743890 minor allele C.


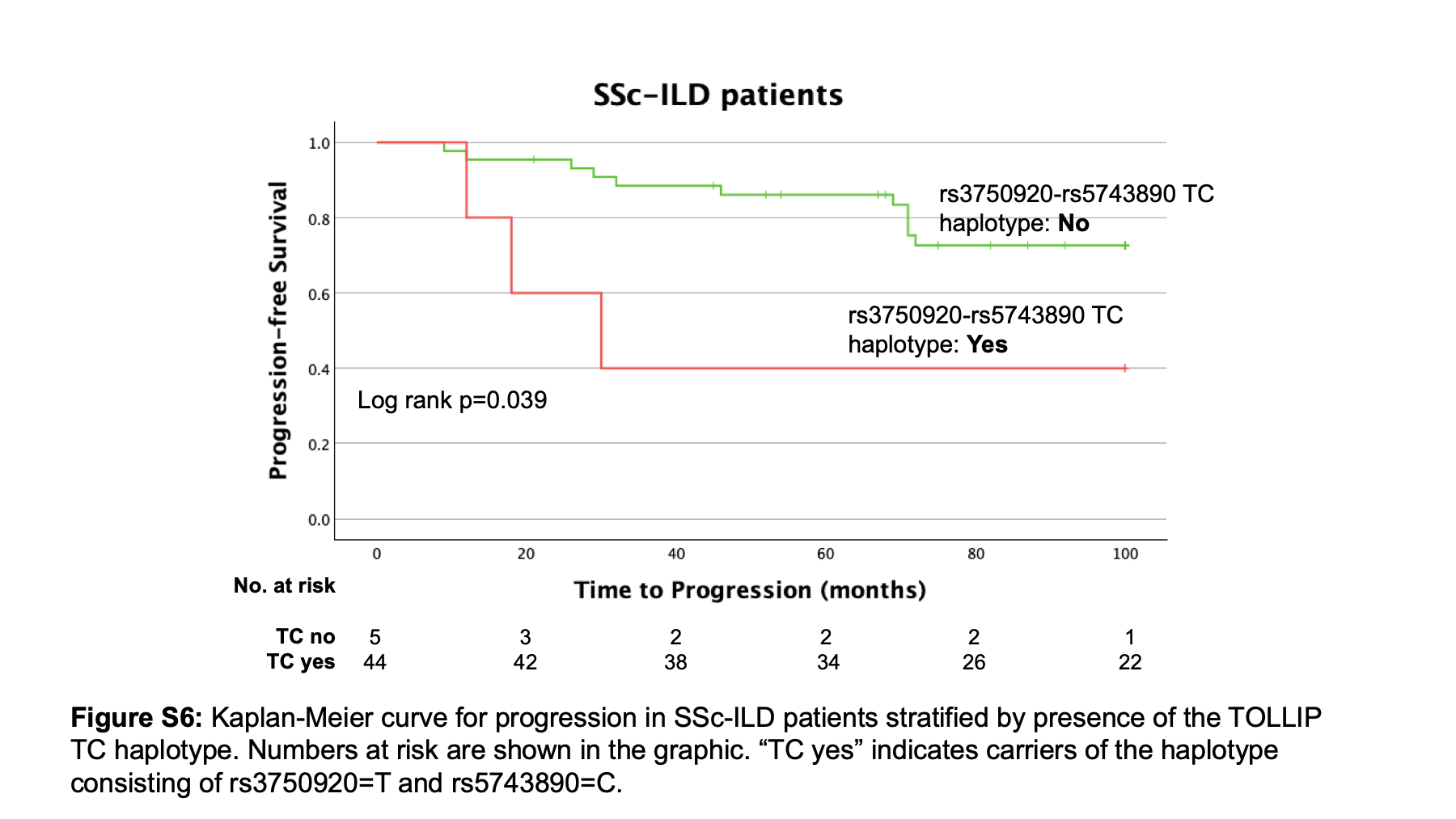


**Figure S7**: Kaplan-Meier curve for progression in SSc-ILD patients stratified by presence of the TOLLIP TC haplotype. “TC yes” indicates carriers of the haplotype consisting of rs3750920=T and rs5743890=C.
